# Unimolecular GLP‐1/Apelin Hybrid Peptides Cause Prominent Appetite Suppression, as Well as Enhancing Insulin Secretion, Beta‐Cell Survival and Glycaemic Regulation

**DOI:** 10.1111/dom.70647

**Published:** 2026-03-16

**Authors:** Ananyaa Sridhar, Ethan S. Palmer, Sarah L. Craig, Elzbieta Krason‐Kidzinska, Nigel Irwin, Finbarr P. M. O'Harte

**Affiliations:** ^1^ Centre for Diabetes, School of Biomedical Sciences Ulster University Coleraine UK

**Keywords:** animal pharmacology, drug development, exenatide, incretin therapy

## Abstract

**Aim:**

To characterise the metabolic benefits of a GLP‐1/apelin hybrid peptide, namely exendin‐4‐linker‐apelin (ELA), and associated acylated forms, including ELA‐Lys^12^(γGluPal), ELA‐Lys^27^(γGluPal) and ELA‐Lys^38^(γGluPal).

**Methods:**

Concentration‐ and receptor‐dependent effects of the peptides on insulin secretion and beta‐cell turnover were investigated using in vitro systems. Subsequent analyses included assessment of food intake and glucose tolerance in normal mice and rats, as well as high‐fat‐fed (HFF) mice.

**Results:**

Enzymatic stability of all ELA peptides was initially confirmed in murine plasma. All peptides significantly augmented insulin secretion from BRIN‐BD11 cells and isolated islets, linked to engagement of both GLP‐1 and apelin receptors, with ELA, ELA‐Lys^12^(γGluPal) and ELA‐Lys^38^(γGluPal) being particularly effective. These hybrid peptides also enhanced beta‐cell proliferation and reduced cytokine‐induced apoptosis. Assessment of effects on feeding behaviour revealed substantial reductions of food intake by ELA, durable for 42 h. Peptide acylation extended the duration of appetite suppressive actions to 63 h. As with in vitro actions, appetite regulatory effects were dependent on engagement of both the GLP‐1 and apelin receptors. Studies in rats suggested that ELA‐induced inhibition of food intake was not linked to malaise. All peptides significantly improved glucose tolerance in lean and HFF mice, indicating enhanced insulin action and glycaemic control. Acylation extended the duration of glucose‐lowering benefits up to 21 h. Effects of ELA on glucose homeostasis appeared to be more reliant on GLP‐1, rather than apelin, receptors.

**Conclusion:**

These studies highlight the clear therapeutic applicability of rationally designed GLP‐1/apelin hybrid molecules for the treatment of obesity and T2DM.

## 1 Introduction

Type 2 diabetes mellitus (T2DM) has now reached pandemic proportions, being driven by obesity and an ageing global population, with estimates indicating that around 800 million adults worldwide currently have T2DM [1]. Even with the wealth of available educational and therapeutic options, metabolic control remains suboptimal for many people living with T2DM [2]. This has motivated intense efforts to identify novel and more effective treatment strategies, highlighted by the comparative success of incretin‐based drugs for obesity and T2DM [3]. As such, exenatide was the first approved glucagon‐like peptide‐1 receptor agonist (GLP‐1RA) [4], and alongside other more recently approved GLP‐1RAs such as liraglutide and semaglutide, provide established glucose‐regulating and body weight reducing actions [5]. Despite these obvious benefits for T2DM, high patient discontinuation rates [6], linked to aspects such as severe side effect profiles or lack of sustained effectiveness [7], emphasise the need for further development in this area.

Recent progress with incretin therapies relates to the evolution of unimolecular co‐agonist peptides, typified by the dual GLP‐1/glucose‐dependent insulinotropic polypeptide (GIP) co‐agonist tirzepatide, that is now approved for both T2DM [8] and obesity [9]. Other comparable dual and triple acting agonists, all with a GLP‐1 backbone, are currently undergoing clinical assessment [10], demonstrating excitement and continued opportunity within this emerging field. In this context, the apelinergic system represents a promising new candidate for multi‐agonist peptide design [11], that has been largely overlooked to date. Apelin is an adipokine and the endogenous ligand for the angiotensin II protein J (APJ) receptor [12], that is widely expressed in key metabolic tissues including the pancreas, adipose, muscle and central nervous system [13]. In keeping with this, apelin exerts potent glucose‐lowering, as well as pancreatic beta‐cell protective effects [14], enhances glucose uptake [15], improves insulin sensitivity [16] and exerts notable benefits on the cardiovascular system [17], representing a complimentary biological action profile to GLP‐1 [3]. Similar to various other circulating hormones, apelin is synthesised as a prepro‐hormone, and then processed intracellularly into various bioactive isoforms [11]. Apelin secretion appears to be stimulated by hypoxia, haemodynamic stress, inflammatory cytokines, as well as circulating insulin, where apelin helps to encourage glucose uptake into skeletal muscle [15]. However, like GLP‐1 [18], the therapeutic utility of native apelin is severely hampered by rapid proteolytic degradation and subsequent peptide inactivation [19]. Consequently, our laboratory has previously demonstrated that rationale modification to the amino acid sequence, and/or acylation, of the smallest bioactive apelin peptide isoform, namely apelin‐13 [20], extends half‐life without compromising bioactivity [21, 22, 23, 24]. The APJ receptor agonist apelin‐13 amide emerged as a promising lead candidate reducing appetite, body weight, circulating glucose and triglycerides, as well as improving glucose tolerance in diet‐induced obese mice [11, 25].

While co‐administration of separate GLP‐1 and apelin peptides is possible, a unimolecular hybrid peptide would offer pharmacokinetic and manufacturing advantages, ensuring fixed‐ratio delivery to target tissues. To this end, we have now constructed a novel GLP‐1/apelin hybrid molecule through fusion of exendin‐4 (1–30) and apelin‐13‐amide. Peptide fusion was achieved through use of an {2‐[2‐aminoethoxy]ethoxy} acetic acid (AEEAc) linker molecule, as previously successfully employed in our, and other, laboratories [26, 27, 28], and the resulting peptide termed exendin‐4‐linker‐apelin (ELA) (Table 1). Exendin‐4 (1–30) was chosen based on previous studies demonstrating effective GLP‐1 receptor agonism of this entity when incorporated within a hybrid peptide sequence [29]. To improve clinical applicability of ELA, related acylated peptides were also synthesised through C‐16 fatty acid conjugation to the free amino group of Lys12, Lys27 or Lys30 via a glutamyl linker, creating ELA‐Lys^12^(γGluPal), ELA‐Lys^27^(γGluPal) and ELA‐Lys^38^(γGluPal), respectively (Table 1). In addition to assessing the enzymatic stability of these peptides, concentration‐ and receptor‐dependent insulinotropic actions and effects on beta‐cell turnover were investigated. Subsequent studies in mice examined dose‐ and time‐dependent effects of all peptides on glucose homeostasis and appetite suppression, with incidence of malaise induction investigated in rats. Our data represent clear early observations that unimolecular peptides with a dual GLP‐1 and APJ receptor activation profile possess strong therapeutic potential for obesity, and associated obesity‐driven forms of diabetes.

**TABLE 1:** Name, amino acid sequence and plasma stability of ELA and related acylated hybrid peptides.

Peptide name	Amino acid sequence	Plasma stability (% intact peptide remaining at 8 h)
Apelin‐13‐amide	Q‐R‐P‐R‐L‐S‐H‐K‐G‐P‐M‐P‐F‐NH_2_	0
Exendin‐4 (1‐30)‐amide	H‐G‐E‐G‐T‐F‐T‐S‐D‐L‐S‐K‐Q‐M‐E‐E‐E‐A‐V‐R‐L‐F‐I‐E‐W‐L‐K‐N‐G‐G‐P‐S‐S‐G‐A‐P‐P‐P‐S‐NH_2_	18 ± 4.6
ELA	H‐G‐E‐G‐T‐F‐T‐S‐D‐L‐S‐K‐Q‐M‐E‐E‐E‐A‐V‐R‐L‐F‐I‐E‐W‐L‐K‐N‐G‐G‐P‐S‐S‐G‐A‐P‐P‐P‐S‐(AEEAc‐AEEAc)‐Q‐R‐P‐R‐L‐S‐H‐K‐G‐P‐M‐P‐F‐NH_2_	100
ELA Lys^12^(γGluPal)	H‐G‐E‐G‐T‐F‐T‐S‐D‐L‐S‐K(γ‐GluPal)‐Q‐M‐E‐E‐E‐A‐V‐R‐L‐F‐I‐E‐W‐L‐K‐N‐G‐G‐P‐S‐S‐G‐A‐P‐P‐P‐S‐(AEEAc‐AEEAc)‐Q‐R‐P‐R‐L‐S‐H‐K‐G‐P‐M‐P‐F‐NH_2_	100
ELA Lys^27^(γGluPal)	H‐G‐E‐G‐T‐F‐T‐S‐D‐L‐S‐K‐Q‐M‐E‐E‐E‐A‐V‐R‐L‐F‐I‐E‐W‐L‐K(γ‐GluPal)‐N‐G‐G‐P‐S‐S‐G‐A‐P‐P‐P‐S‐(AEEAc‐AEEAc)‐Q‐R‐P‐R‐L‐S‐H‐K‐G‐P‐M‐P‐F‐NH_2_	100
ELA Lys^38^(γGluPal)	H‐G‐E‐G‐T‐F‐T‐S‐D‐L‐S‐K‐Q‐M‐E‐E‐E‐A‐V‐R‐L‐F‐I‐E‐W‐L‐K‐N‐G‐G‐P‐S‐S‐G‐A‐P‐P‐P‐S‐(AEEAc‐AEEAc)‐Q‐R‐P‐R‐L‐S‐H‐K(γ‐GluPal)‐G‐P‐M‐P‐F‐NH_2_	100

*Note:* Peptide name and amino acid sequences depicted using standard single‐letter notation, where AEEAc is {2‐[2‐aminoethoxy]ethoxy}acetic acid and γ‐GluPal represents a C‐16 fatty acid conjugated to the free amino group of respective Lys residue via a glutamyl linker. For plasma stability, peptides (50 μg) were incubated with overnight fasted murine plasma (5 μL) for 0–8 h and reactions terminated by the addition of trifluoroacetic acid (10% v/v). Degradation profiles were then analysed using RP‐HPLC with absorbance at 214 nm and HPLC peaks of interest collected for subsequent MALDI‐MS analysis.

## 2 Methods

### 2.1 Peptides

All peptides were purchased from Synpeptide (Shanghai, China) at 95% purity and characterisation was further corroborated in‐house using HPLC and MALDI‐TOF MS analysis, as previously described [30]. The names and amino acid sequence of all peptides are provided within Table 1.

### 2.2 Plasma Degradation

ELA and related acylated peptides (50 μg) were incubated at 37°C for 0 or 8 h with 5 μL of overnight fasted murine plasma. Degradation reactions were terminated by addition of 10 μL 10% (vol/vol) trifluoroacetic acid/water solution. Reaction mixes were separated by reverse phase HPLC (Phenomenex C‐18 analytical column, 250 × 4.6 mm^2^) with UV absorbance detection at 214 nm (ThermoQuest SpectraSystem UV2000 detector). HPLC peaks of interest were collected and analysed by MALDI‐TOF MS on a Perseptive Biosystems Voyager‐DE Biospectrometry (Hertfordshire, UK).

### 2.3 In Vitro and Ex Vivo Insulin Secretion

Effects of test peptides on in vitro insulin secretion were examined using BRIN‐BD11 beta‐cells, whose characteristics have been reported previously [31]. Notably, BRIN‐BD11 cells mimic the glucose sensitivity and overall secretory performance of other beta‐cell lines and isolated islets, representing an accepted and well‐characterised model for beta‐cell function studies [32]. Cells were seeded (150 000/well) into 24‐well plates (Nunc, Roskilde, Denmark) and allowed to attach overnight at 37°C. Following 40 min pre‐incubation (1.1 mmol/L glucose; 37°C), cells were incubated (20 min; 37°C) in the presence of 5.6 mmol/L glucose with a range of test peptide concentrations (10^−12^–10^−6^ M). In an additional experimental series, ELA peptides were incubated in the absence or presence of the GLP‐1R antagonist exendin‐4 (9–39) [33] and/or the APJ receptor antagonist (Val^13^)apelin‐13 [22] to help determine receptor specificity. Importantly, both exendin‐4 (9–39) and (Val^13^)apelin‐13 have been utilised previously in our laboratory and shown not to interfere with any of the metabolic indicators assessed in the current study [22, 34]. After a 20 min incubation, buffer was removed from each well and aliquots stored at −20°C prior to the determination of insulin by radioimmunoassay [35]. Furthermore, the impact of ELA peptides on insulin secretion (*n* = 4; 60 min incubation; 10^−8^ and 10^−6^ M) from murine islets (12‐week‐old C57BL/6 mice) isolated by standard collagenase digestion was also examined, as described previously [36].

### 2.4 Beta‐Cell Proliferation and Apoptosis

The impact of ELA and acylated analogues on BRIN‐BD11 beta‐cell proliferation (40 000 cells per well) was assessed using the Ki‐67 primary antibody (Ab15580, AbCam, Cambridge, UK) and the Alexa Fluor 594 secondary antibody, as described previously [37]. For apoptosis studies, cellular stress was induced through incubation of BRIN‐BD11 cells with a cytokine cocktail (IL‐1beta 100 U/mL, IFN‐γ 20 U/mL, TNF‐α 200 U/mL), and the rate of apoptosis was monitored by TUNEL staining (Fluorescein, Roche Diagnostics, Burgess Hill, UK), as described previously [37]. For both proliferation and apoptosis, effects were visualised using a fluorescence microscope (Olympus system microscope, model BX51; Southend‐on‐Sea, UK) and a DP70 camera adapter system using DAPI (350 nm), TRITC (594 nm) and FITC (488 nm) filters, alongside an Olympus XM10 camera. For quantification, the cell‐counter function within ImageJ Software Version 1.54 (National Institutes of Health (NIH), Bethesda, MD, USA) was employed to establish the number of positively stained Ki‐67 or TUNEL beta‐cells, as appropriate. All data were presented as a percentage of the total number of cells investigated.

### 2.5 Animals

Animal studies were conducted using adult male C57BL/6 mice or Sprague Dawley rats (14–16 weeks of age, Envigo, UK) housed individually in an air‐conditioned room at 22°C ± 2°C with a 12 h light and dark cycle. Rodents had *ad libitum* access to drinking water and standard rodent maintenance diet (10% fat, 30% protein and 60% carbohydrate; Trouw Nutrition, Northwick, UK) or a high‐fat diet (45% fat, 20% protein and 35% carbohydrate; Special Diets Service, Essex, UK), as appropriate, unless otherwise stated. All procedures were performed in compliance with the UK Animal Scientific Procedures Act 1986, covered under a UK Home Office Animal project licence (UK Home Office licence certificate PPL2902 approved on 26 April 2021), and approved by the Ulster Animal Welfare and Ethical Review Body (AWERB) committee.

### 2.6 In Vivo Feeding Studies

Experiments were performed in mice habituated to a daily feeding regime of 3 h/day, from 10:00 to 13:00 h. These mice were subject to a progressive reduction in the daily feeding period over 3 weeks at 12 weeks of age, as detailed previously [22, 38]. Mice were maintained on this 3 h/day feeding regimen throughout the study period, and were able to consume their daily calorie requirement within this feeding window with no obvious negative effect on behaviour or physiological responses [22, 38]. For experimentation, animals received an intraperitoneal (i.p.) injection of saline vehicle (0.9% wt/vol NaCl) or test peptide (0.5–25 nmol/kg bw) immediately prior to the 3 h food intake window, and consumption of food recorded at 30 min intervals over the 3 h period. In a separate series, test peptides (2.5 nmol/kg bw) were injected concomitantly with exendin‐4 (9–39) and/or (Val^13^)apelin‐13 (both at 25 nmol/kg bw). In another set of experiments in this habituated mouse model, peptides (25 nmol/kg bw) were administered 1, 6, 12, 21, 42, 63 and 84 h prior to the 3 h window of food intake monitoring. For the 42, 63 and 84 h delayed injections, mice remained habituated to the daily feeding regime of 3 h/day, on the day(s) prior to food intake monitoring after peptide administration. Finally, to test for potential malaise, kaolin clay intake was monitored in overnight fasted Sprague Dawley rats (*n* = 6) following i.p. administration of ELA (25 nmol/kg bw). Rats were employed for the detection of malaise, as mice are much less responsive to the kaolin clay assay.

### 2.7 In Vivo Glucose Homeostasis Studies

Freely fed mice, fasted overnight, were employed to examine the impact of ELA and related peptides on glucose homeostasis. Blood glucose was measured immediately before and at intervals after i.p. administration of glucose alone (18 mmol/kg bw) or in combination with test peptides (25 nmol/kg bw). In addition, test peptides (25 nmol/kg bw) were administered 8, 12, 21 and 36 h prior to the glucose challenge to investigate duration of biological effects. In a separate series, the glucose homeostatic efficacy of ELA was examined in high fat fed (HFF) mice. These mice were transferred to the 45% high fat diet at 5 weeks of age and maintained on that diet until experimentation at 14–16 weeks of age. To probe receptor dependency of ELA effects, the impact of combined injection with (Val^13^)apelin‐13 and/or exendin‐4 (9–39) (all at 25 nmol/kg bw) was also investigated in HFF mice. For all experiments, blood samples were collected from the cut tip on the tail vein of conscious mice at the time points indicated in the Figures. Blood glucose was then directly measured using a hand‐held Ascencia Contour blood glucose metre (Bayer Healthcare, Newbury, Berkshire, UK).

### 2.8 Statistical Analysis

Statistical analyses were performed using GraphPad PRISM software (Version 10.3.0). Values are expressed as mean ± SEM. Data were assessed for normality using the Shapiro–Wilk test and for homogeneity of variance using the Brown‐Forsythe test prior to parametric analysis. For repeated‐measures analyses, sphericity was evaluated and the Geisser–Greenhouse correction applied to the F‐statistic where necessary. Comparative analyses between groups were carried out using One‐way ANOVA or Two‐way ANOVA, as appropriate, with a Bonferroni post hoc test. In vivo experimental sample size (*n* = 6–8) was calculated based on previous experience with the experimental systems employed, assuming a two‐sided significance level of α = 0.05 and power (1 − β) = 0.8. The difference between groups was considered significant if *p* < 0.05.

## 3 Results

### 3.1 ELA and Related Acylated Analogues, Directly Stimulate Insulin Secretion, an Effect That Is Dependent on Both GLP‐1 and APJ Receptor Activation

**FIGURE 1:**
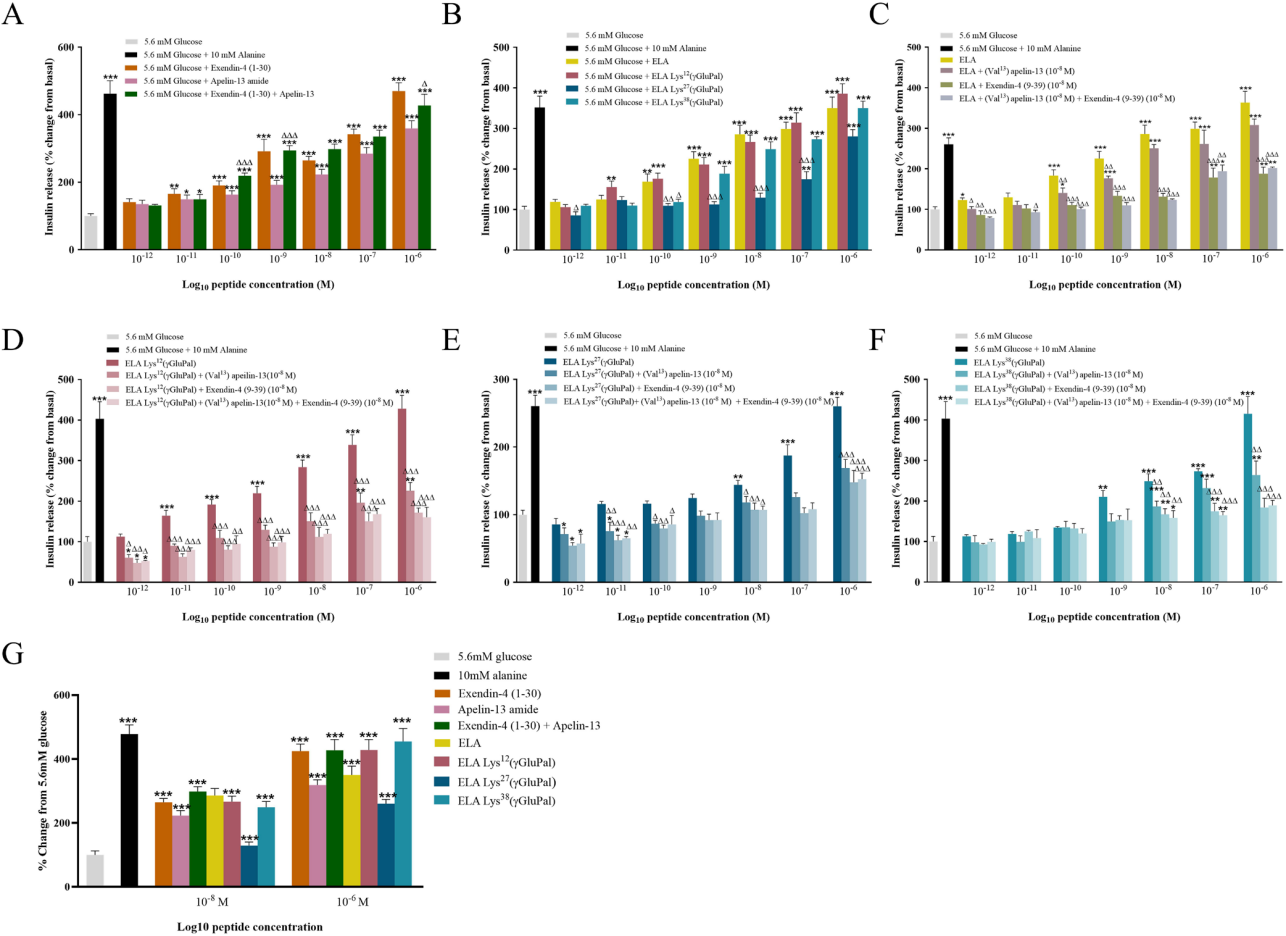
Dose‐dependent effects of ELA, and related acylated analogues, on insulin secretion from BRIN‐BD11 cells and mouse islets. (A, B) BRIN‐BD11 cells were incubated for 20 min with test peptides (10^−12^ to 10^−6^ M) in the presence of 5.6 mM glucose. (C–F) Insulin secretory effects of (C) ELA (D) ELA Lys^12^(γGluPal), (E) ELA Lys^27^(γGluPal) and (F) ELA Lys^38^(γGluPal) (10^−12^ to 10^−6^ M, 20 min) in combination with GLP‐1 and/or APJ receptor antagonists (each at 10^−8^ M). (G) Murine islets were incubated for 60 min with test peptides (10^−8^ or 10^−6^ M M) in the presence of 5.6 mM glucose. For all experiments insulin concentrations were measured by RIA. All values represent mean ± SEM (A–F *n* = 8, G *n* = 4). *p < 0.05, **p < 0.01 and ***p < 0.001 compared to respective glucose control. ^∆^ *p* < 0.05, ^∆∆^ *p* < 0.01 and ^∆∆∆^ *p* < 0.001 compared to (A) apelin‐13‐amide alone, (B,C) ELA and (D‐F) respective acylated peptide.

As expected, apelin‐13‐amide and exendin‐4 (1–30) significantly (F(18,115) = 29.14; *p* < 0.05–0.001) augmented insulin secretion in a dose‐dependent manner from BRIN‐BD11 cells at concentrations ranging from 10^−11^ to 10^−6^ M when compared to 5.6 mM glucose alone control (Figure 1A). In keeping with this, combined incubation of apelin‐13‐amide and exendin‐4 (1–30) also augmented insulin secretion, being significantly more efficacious (F(9,60) = 36.39; *p* < 0.05–0.001) than apelin‐13‐amide alone at 10^−10^, 10^−9^ and 10^−6^ M concentrations (Figure 1A). Encouragingly, ELA, ELA‐Lys^12^(γGluPal), ELA‐Lys^27^(γGluPal) and ELA‐Lys^38^(γGluPal) also demonstrated prominent (F(29,192) = 31.69; *p* < 0.05–0.001) insulin secretory actions, with ELA and ELA‐Lys^12^(γGluPal) being particularly effective in this regard (Figure 1B). In terms of receptor dependency, blockade of GLP‐1 receptors, either alone or in combination with APJ receptor antagonism, significantly (F(22,102) = 23.11; *p* < 0.05–0.001) inhibited ELA‐induced insulin secretion (Figure 1C). Use of the APJ receptor antagonist alone also partially annulled ELA‐mediated effects, but only at selected concentrations (10^−12^, 10^−10^ and 10^−9^ M) and not to the same degree as GLP‐1 receptor inhibition (Figure 1C). Interestingly, both exendin‐4 (9–39) and (Val^13^)apelin‐13, either alone or in combination, prominently (F(29,132) = 25.26; F(29,140) = 27.83; F(29,123) = 15.1, respectively; *p* < 0.05–0.001) reduced insulin release evoked by all three of the fatty acid derived ELA hybrid peptides (Figure 1D–F). Initial studies in isolated murine islets confirmed insulinotropic effectiveness (F(16,110) = 34.45; *p* < 0.001) of apelin‐13‐amide, exendin‐4 (1–30), ELA and related acylated analogues at 10^−8^ and 10^−6^ M, although efficacy of ELA‐Lys^27^(γGluPal) was somewhat less obvious (Figure 1G), in keeping with observations in BRIN‐BD11 cells (Figure 1B).

### 3.2 ELA, ELA‐Lys^12^(γGluPal) and ELA‐Lys^38^(γGluPal) Augment Beta‐Cell Growth and Survival

**FIGURE 2:**
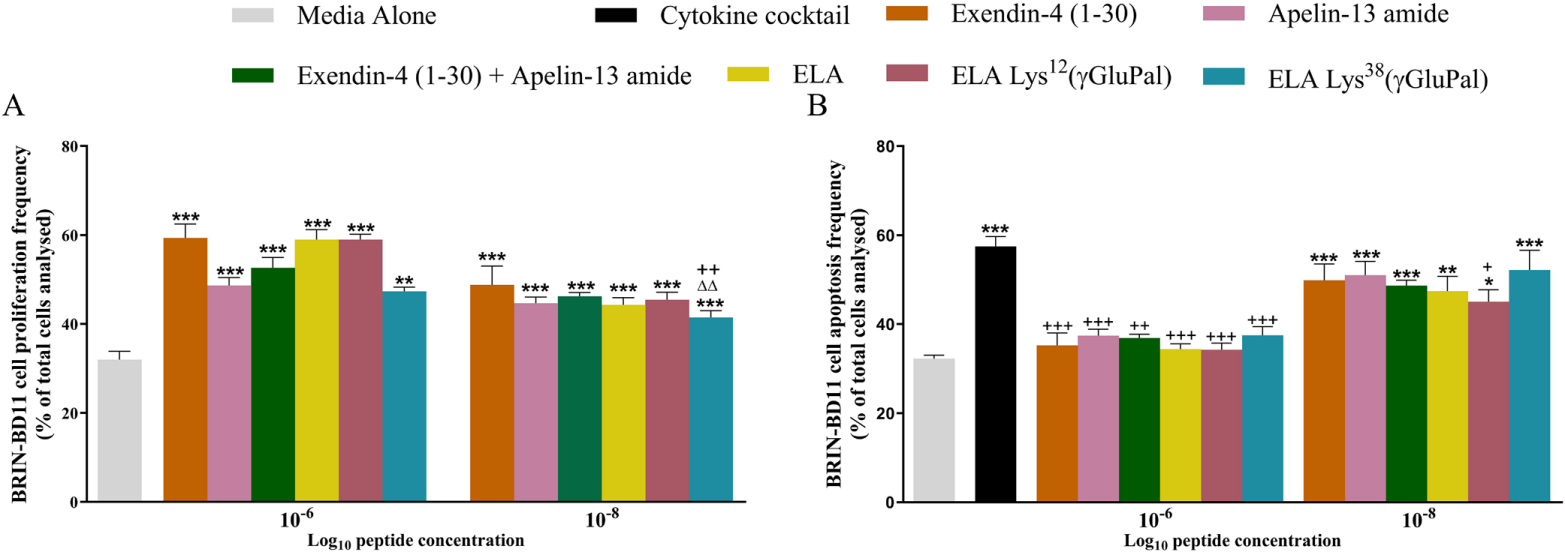
Effect of ELA, ELA Lys^12^(γGluPal) and ELA Lys^38^(γGluPal) on BRIN‐BD11 beta‐cell proliferation and protection against cytokine‐induced apoptosis. (A) BRIN‐BD11 cells were incubated overnight (18 h) with test peptides (each at 10^−8^ and 10^−6^ M). Proliferation frequency was measured using Ki‐67 immunocytochemistry. (B) TUNEL‐positive apoptotic cells were assessed following 2 h exposure to a cytokine cocktail (IL‐1β 100 U/mL, IFN‐γ 20 U/mL, TNF‐α 200 U/mL) with or without co‐culture in the presence of test peptides (each at 10^−8^ and 10^−6^ M). All values represent mean ± SEM (*n* = 3). *p < 0.05, **p < 0.01 and ***p < 0.001 compared to respective media control. ^∆∆^ *p* < 0.01 compared to 10^−6^ M ELA. ^+^ *p* < 0.05, ^++^ *p* < 0.01 and ^+++^ *p* < 0.001 compared to (A) ELA Lys^12^(γGluPal) or (B) cytokine cocktail.

ELA, and the related acylated peptides that were examined, augmented BRIN‐BD11 beta‐cell proliferation at both 10^−6^ and 10^−8^ M when compared to media control (Figure 2A). However, at 10^−8^ M, ELA‐Lys^38^(γGluPal) was significantly (F(12, 75) = 15.45; *p* < 0.01) less effective than ELA and ELA‐Lys^12^(γGluPal) (Figure 2A). The cytokine cocktail increased (F(13, 77) = 10.24; *p* < 0.001) BRIN‐BD11 cellular apoptosis rates, but all peptides significantly (F = (12, 72) = 8.3; *p* < 0.001) attenuated this detrimental effect at 10^−6^ M, returning apoptosis rates similar to those observed with media alone (Figure 2B). However, at a concentration of 10^−8^ M, only ELA‐Lys^12^(γGluPal) reduced (F (2, 19) = 54.41; *p* < 0.05) apoptosis when compared to the cytokine cocktail alone (Figure 2B).

### 3.3 ELA and Related Acylated Analogues, Dose‐Dependently Suppress Appetite Without Inducing Obvious Malaise, and Effects Are Partially Dependent on GLP‐1 and APJ Receptor Activation

**FIGURE 3:**
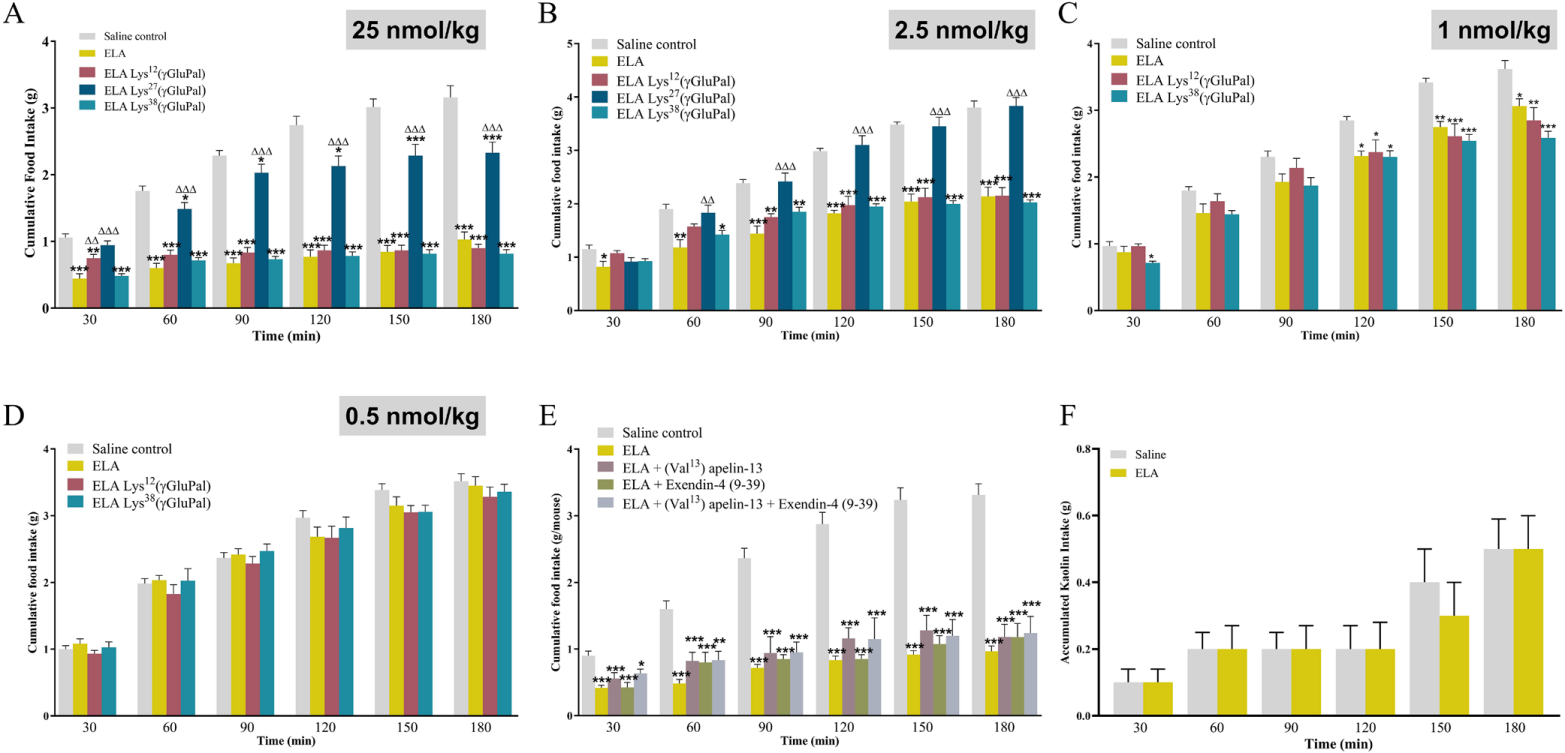
Effects of ELA, and related acylated analogues, on food intake in mice. Cumulative food intake was measured at 30, 60, 90, 120, 150 and 180 min in mice trained to feed within a daily 3 h window. (A–D) Mice received intraperitoneal (i.p.) injections of either saline vehicle (0.9% wt/vol NaCl), ELA, ELA Lys^12^(γGluPal), ELA Lys^27^(γGluPal) or ELA Lys^38^(γGluPal) at doses of (A) 25, (B) 2.5, (C) 1 or (D) 0.5 nmol/kg bw. (E) Cumulative food intake following i.p. injection of saline vehicle or ELA (25 nmol/kg bw), alone and in combination with GLP‐1 and/or APJ receptor antagonists (each at 25 nmol/kg bw). (F) Accumulated kaolin clay intake following i.p. injection of ELA (25 nmol/kg bw) in overnight fasted rats. All values represent mean ± SEM (A‐E *n* = 8, F *n* = 6). *p < 0.05, **p < 0.01 and ***p < 0.001 compared to respective saline control. ^∆∆^ *p* < 0.01 and ^∆∆∆^ *p* < 0.01 compared to ELA.

At 25 nmol/kg, ELA and all related acylated analogues reduced (F(29,168) = 66.29; *p* < 0.01–0.001) food intake in mice (Figure 3A). ELA, ELA‐Lys^12^(γGluPal) and ELA‐Lys^38^(γGluPal) were particularly effective in this regard, whereas ELA‐Lys^27^(γGluPal) was significantly (F(12, 78) = 38.49; *p* < 0.001) less effective than the parent ELA hybrid peptide (Figure 3A). Indeed, at a dose of 2.5 nmol/kg, ELA‐Lys^27^(γGluPal) was devoid of appetite suppressive actions, whereas ELA, ELA‐Lys^12^(γGluPal) and ELA‐Lys^38^(γGluPal) retained effectiveness (F(29, 120) = 53.86; *p* < 0.05–0.001) to inhibit food intake (Figure 3B). At 1 nmol/kg, these three latter peptides still displayed appetite suppressing actions (F(23,149) = 45.64; *p* < 0.05–0.001), but only at the later time points and with a decreased magnitude of effect when compared to either 25 or 2.5 nmol/kg peptide doses (Figure 3C). It follows that the ability of all ELA peptides to regulate feeding behaviour was absent at a dose of 0.5 nmol/kg (Figure 3D). The appetite inhibitory effects of ELA, ELA‐Lys^12^(γGluPal) and ELA‐Lys^38^(γGluPal) were demonstrated to be partially dependent on engagement of both GLP‐1 or APJ receptors, with individual or combined receptor inhibition leading to numerically elevated, but non‐significant, increases in food intake (Figure 3E). To investigate if ELA‐mediated appetite suppression was linked to nausea and general gastrointestinal discomfort, a kaolin clay ingestion test was conducted in rats. At a dose of 25 nmol/kg, ELA did not increase kaolin clay intake beyond the minimal amount consumed by saline‐treated control rats, that is linked to initial inquisitory exploration (Figure 3F).

### 3.4 ELA, ELA‐Lys12(γGluPal) and ELA‐Lys38(γGluPal) Possess Persistent Appetite Suppressive Actions

**FIGURE 4:**
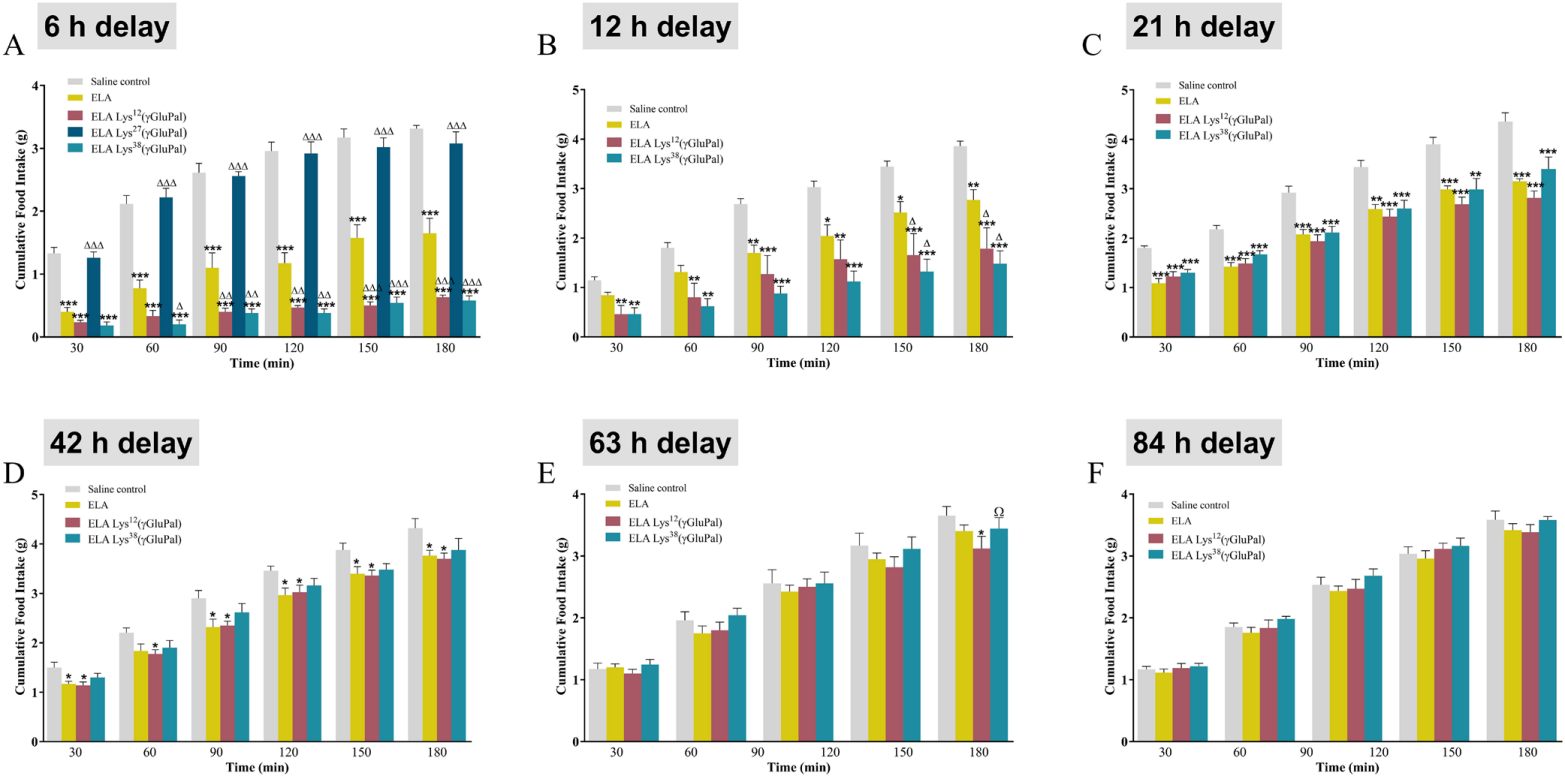
Time‐dependent effects of ELA, and related acylated analogues, on food intake in mice. Cumulative food intake was measured at 30, 60, 90, 120, 150 and 180 min in mice trained to feed within a daily 3 h window. (A–D) Mice received intraperitoneal (i.p.) injections of either saline vehicle (0.9% wt/vol NaCl), ELA, ELA Lys^12^(γGluPal), ELA Lys^27^(γGluPal) or ELA Lys^38^(γGluPal), as appropriate, at (A) 6, (B) 12, (C) 21, (D) 42, (E) 63 or (F) 84 h previously. All values represent mean ± SEM (*n* = 8). *p < 0.05, **p < 0.01 and ***p < 0.001 compared to respective saline control. ^∆^ *p* < 0.05, ^∆∆^ *p* < 0.01 and ^∆∆∆^ *p* < 0.001 compared to ELA.

Six hours after administration, ELA‐Lys^27^(γGluPal) lacked any obvious appetite suppressing actions, whereas ELA, and particularly ELA‐Lys^12^(γGluPal) as well as ELA‐Lys^38^(γGluPal), remained highly effective (F(29, 114) = 73.38; *p* < 0.05–0.001) in this regard (Figure 4A). Similar observations were made following injection of the peptides 12 h previously, with ELA‐Lys^12^(γGluPal) and ELA‐Lys^38^(γGluPal) displaying some superiority (F(23,132) = 15.8; *p* < 0.05) over ELA (Figure 4B). Interestingly, when administered 21 h previously, ELA was equally as effective as ELA‐Lys^12^(γGluPal) and ELA‐Lys^38^(γGluPal) (F(23,114) = 42.43; *p* < 0.01–0.001) in terms of inhibiting food intake (Figure 4C). Following a 42 h delay, both ELA and ELA‐Lys^12^(γGluPal) were still capable of inducing significant appetite suppression (F(23, 99) = 48.63; *p* < 0.05), whereas ELA‐Lys^38^(γGluPal) was not (Figure 4D). Only ELA‐Lys^12^(γGluPal) was able to inhibit appetite when injected 63 h previously, and this effect was modest (F(19, 94) = 28.58; *p* < 0.05) and only observed at the 180 min observation point (Figure 4E). All peptide bioactivity was lost when administered 84 h prior to monitoring of food intake (Figure 4F).

### 3.5 ELA and Related Acylated Analogues, Improve Glucose Homeostasis in Normal and High Fat Fed Mice

**FIGURE 5:**
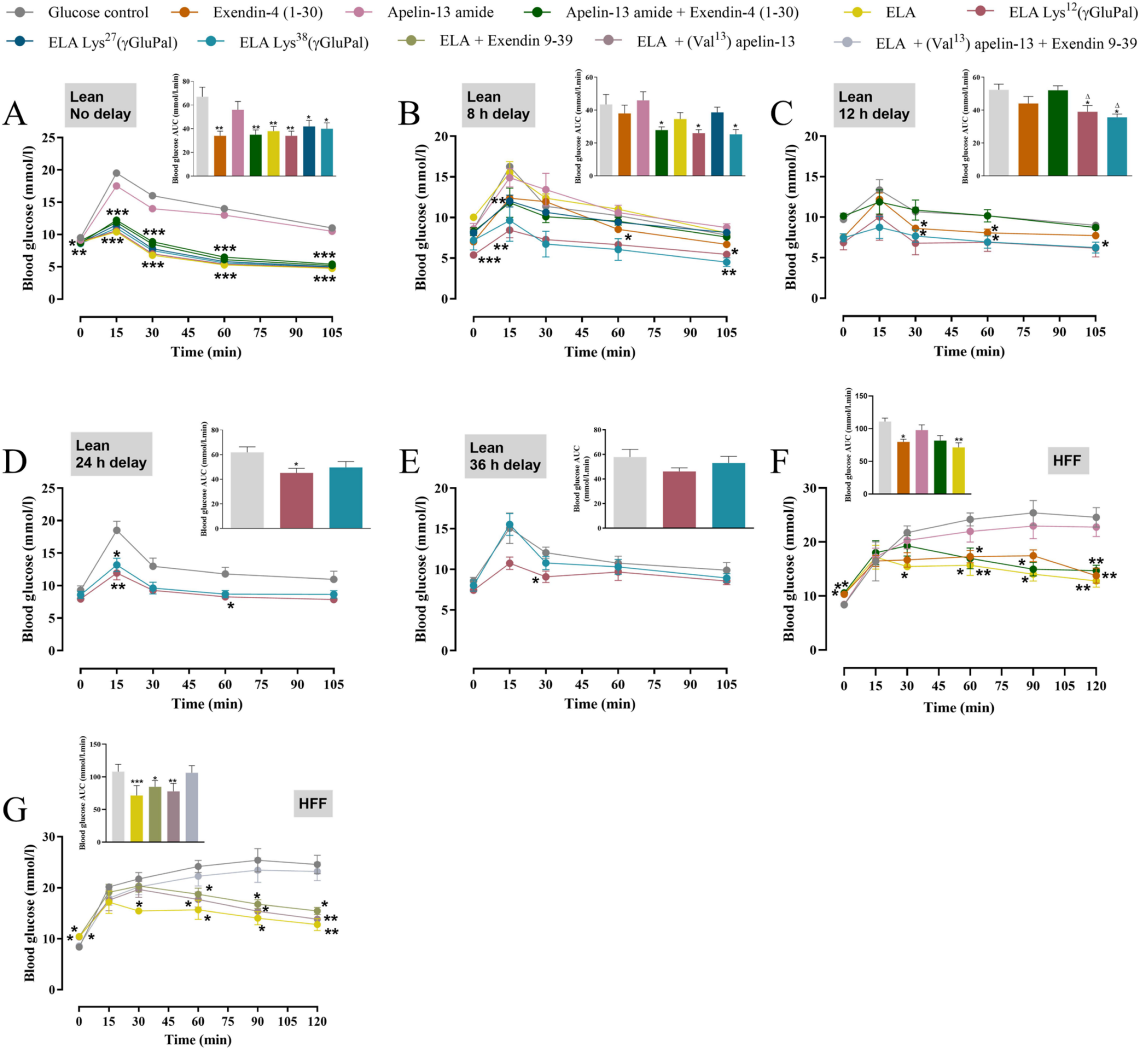
Effects of ELA, and related acylated analogues, on glucose tolerance in lean and HFF mice. Blood glucose concentrations were measured at baseline (0 min) and at 15, 30, 60, 105 and 120 min, as appropriate, following an intraperitoneal (i.p.) glucose load (18 mmol/kg bw) in overnight fasted (A–E) normal or (F, G) HFF mice. (A–E) Mice were injected with saline vehicle (0.9% wt/vol NaCl) or test peptides (25 nmol/kg bw) at (A) 0, (B) 8, (C) 12, (D) 21 and (E) 36 h prior to the glucose challenge. (F) HFF mice received glucose control (18 mmol/kg bw) alone or in combination with test peptide (25 nmol/kg bw), as well as (G) ELA (25 nmol/kg bw) alone or in combination with GLP‐1 and/or APJ receptors antagonists (each at 25 nmol/kg bw). For all studies, area under the curve (AUC) for blood glucose is also shown. All values are presented as mean ± SEM (*n* = 6 mice per group). *p < 0.05, **p < 0.01 and ***p < 0.001 compared to respective glucose alone control. ^Δ^ *p* < 0.05 compared to combined apelin‐13‐amide plus exendin‐4 (1–30) administration.

There was a clear augmentation (F (7, 39) = 5.062; *p* < 0.05–0.01) of glucose‐lowering effects following concurrent administration of ELA, or related acylated analogues, together with glucose to normal mice, when compared to glucose alone control (Figure 5A). When administered 8 h prior to a glucose challenge, the combination of apelin‐13‐amide and exendin‐4 (1–30), as well as ELA‐Lys^12^(γGluPal) and ELA‐Lys^38^(γGluPal), significantly reduced (F(7, 32) = 3.829; *p* < 0.05) overall 0–105 min glucose AUC values, despite no significant alteration of blood glucose levels at any of the individual time points examined (Figure 5B). At 12 h post‐administration, only ELA‐Lys^12^(γGluPal) and ELA‐Lys^38^(γGluPal) retained glucose homeostatic actions following a glucose challenge (F(4, 20) = 5.135; *p* < 0.05) when compared to both glucose control and combined injection of the parent peptides, but this effect was only evident when analysing 0–105 min glucose AUC values (Figure 5C). When injected 24 h prior to the glucose challenge, both ELA‐Lys^12^(γGluPal) and ELA‐Lys^38^(γGluPal) lowered (F(8, 60) = 2.65; *p* < 0.05–0.01) blood glucose at 15, 30, and 60 min post glucose injection, but only ELA‐Lys^12^(γGluPal) also reduced (F(2, 12) = 3.986; *p* < 0.05) AUC glucose values (Figure 5D). There were no discernible glucose lowering actions of either ELA‐Lys^12^(γGluPal) or ELA‐Lys^38^(γGluPal) when administered 36 h previously (Figure 5E). In HFF mice, exendin‐4 (1–30) and ELA retained glucose lowering actions, but apelin‐13‐amide either alone or in combination with exendin‐4 (1–30), was unable to alter circulating glucose levels when injected conjointly with glucose in this model (Figure 5F). Interestingly, neither exendin‐4 (9–39) nor (Val^13^)apelin‐13 alone were able to inhibit the prominent (F (4, 20) = 9.675; *p* < 0.05) glucose regulatory actions of ELA, but combined injection of exendin‐4 (9–39) and (Val^13^)apelin‐13 did prevent ELA related reductions in blood glucose (Figure 5G).

## 4 Discussion

The present study provides clear evidence that combined GLP‐1 and APJ receptor activation is a highly effective strategy for the treatment of obesity and related T2DM. Given some of the key clinical issues with currently prescribed incretin mimetics that relate to GIT side effects [39] alongside lack of sustainable metabolic benefits [40], the ability of ELA and associated acylated analogues to impart substantial and sustainable glucose‐lowering and appetite suppressive actions, in the absence of any obvious malaise, is highly encouraging.

In the in vitro setting, ELA and related acylated analogues were able to prominently amplify pancreatic beta‐cell function. Interestingly, the insulinotropic efficacy of ELA appeared to be superior to that of combined incubation with the parent peptides. Thus, the hybrid peptide may preferentially activate GLP‐1 and APJ intracellular signalling pathways that encourage insulin secretion, in a manner that is superior to either native GLP‐1 and apelin. Indeed, as documented with other dual and triple acting incretin‐based compounds [41], biased agonism by hybrid peptides can favour receptor recycling over desensitisation [42], and could be important in this regard. Notably, GLP‐1 is recognised to activate Gs‐coupled cAMP and PKA‐dependent beta‐cell systems that prime insulin granules for exocytosis [43], whereas APJ engages calcium‐dependent PI3K‐Akt and ERK‐mediated pathways [44]. The convergence of these signalling pathways also likely underlies the amplified insulin secretory responses observed, providing good credence for the current hybrid peptide design strategy. This theory is reinforced through the use of specific GLP‐1 and APJ receptor antagonists, confirming that blockade of either receptor attenuated insulin secretion, but simultaneous receptor inhibition largely abolished responses. However, related receptor interactions and downstream cell signalling pathways have not yet been investigated. This would represent further mechanistic studies which fall outside the scope of the current study, that predominantly focuses on characterising translatability of biological effects of the GLP‐1/apelin hybrid peptides. It is highly encouraging that, despite fatty acid derivation and presence of albumin within the test buffers [45], both ELA‐Lys^12^(γGluPal) and ELA‐Lys^38^(γGluPal) possessed impressive insulinotropic bioactivity in BRIN‐BD11 cells and islets. Importantly, earlier observations with GLP‐1 [46] and apelin peptides [22] confirm physiologically relevant glucose‐dependency of these actions, meaning risk of hypoglycaemia should be minimal.

The differential activity of the acylated ELA analogues is striking and clearly illustrates how hybrid peptide architecture influences biological activity and therapeutic applicability. As such, the structures of ELA‐Lys^12^(γGluPal), ELA‐Lys^27^(γGluPal) and ELA‐Lys^38^(γGluPal) differ only by site of acylation. Yet, ELA‐Lys^12^(γGluPal) and ELA‐Lys^38^(γGluPal) retained strong bioactivity within the in vitro and ex vivo systems employed that translated to prominent in vivo efficacy in rodents, while ELA‐Lys^27^(γGluPal) exhibited markedly reduced bioactivity. Thus, as observed in our own and other laboratories [22, 47] site of lipidation can strongly influence receptor binding, signalling bias and biological potency. Additional studies in beta‐cells with ELA and the two acylated peptides that retained obvious bioactivity, namely ELA‐Lys^12^(γGluPal) and ELA‐Lys^38^(γGluPal), revealed enhanced proliferation and reductions of stress‐induced beta‐cell apoptosis, confirming protective actions in addition to direct promotion on insulin secretion. This is particularly encouraging given the progressive nature of beta‐cell loss and dysfunction that drives T2DM [48] and aligns well with established benefits of GLP‐1 and APJ receptor engagement on overall beta‐cell health [24, 49].

Our in vivo investigations provided an important extension of these cellular effects, confirming full translation and sustainability of the biological action profile of the ELA hybrid peptides. In keeping with prominent effects on beta‐cells and enhanced enzymatic stability of all ELA‐related peptides, the acylated analogues, particularly ELA‐Lys^12^(γGluPal) and ELA‐Lys^38^(γGluPal), provoked persistent improvements in glucose handling when administered up to 24 h before a glucose challenge, corroborating extension of biological half‐life through simple acylation [50]. The enhanced glucose homeostatic actions of the ELA peptides likely reflect augmented insulin secretion, as observed in BRIN‐BD11 beta‐cells and isolated islets, as well as the ability of GLP‐1 and APJ receptor activation to promote glucose uptake in peripheral tissues [16, 49]. It is interesting that apelin‐13 amide was highly susceptible to enzymatic breakdown, whereas ELA, that contains the apelin‐13 amide sequence within its C‐terminal portion, was completely resistant to enzyme degradation. It follows that integration of apelin‐13 amide alongside exendin‐4 (1–30) and two AEEAc linker molecules in ELA protects against natural peptidase action, likely through stearic hindrance as suggested for other hybrid peptides [28], which fits well with the extended bioactive profile of ELA in the current setting. In addition, in high‐fat‐fed mice, while combined administration of the parent peptides lacked clear glucose homeostatic activity in keeping with disrupted endocrine function in this mouse model [51], exendin‐4 (1–30) alone and ELA demonstrated moderate, but observable, glucose lowering efficacy. This points towards the maintained effectiveness of GLP‐1 receptor activation pathways in obesity and T2DM [52], as well as the possible differential downstream signalling selectivity of the ELA peptides [42]. In agreement with this unimolecular co‐agonist approach, glucose‐lowering efficacy of ELA persisted in the face of individual receptor blockade, but not with combined receptor inhibition.

Further to this, ELA produced robust reductions in food intake in mice at doses as low as 1 nmol/kg, reflecting strong engagement of GLP‐1 and APJ receptor‐mediated satiety pathways [21, 22, 25]. Intriguingly, the anorectic effect of the peptides appeared to be somewhat delayed at reduced doses, possibly reflecting rate of peptide passage through the blood‐brain barrier. Remarkably, ELA exhibited a pronounced protraction of anorectic effect, up to 42 h in duration, that was only moderately extended by peptide acylation. The prolonged appetite suppressive effects of ELA, that are not fully replicated in terms of sustainability of glucose‐lowering actions, may therefore be linked to site of action. Thus, ELA‐mediated effects of energy intake are anticipated to be related to modulation of hypothalamic pathways, where GLP‐1 and APJ receptors are expressed [53, 54]. It follows that passage of peptides through the blood–brain barrier and subsequent retention in this setting, as well as circulating half‐life, could determine overall impact on energy homeostasis. That said, all investigations in rodents in the current setting related to single peptide injections. More detailed studies employing sustained administration schedules are required to assess full therapeutic applicability and associated underlying mechanisms, that would include direct comparison with clinically approved GLP‐1 mimetics or related dual‐acting drugs such as tirzepatide. Notably, ELA‐Lys^27^(γGluPal) had less obvious impact on appetite, reinforcing that site of acylation is critical for preservation of bioactivity [55], despite full enzymatic stability and probable extension of pharmacokinetic profile of this hybrid peptide.

Overall, these findings leave ELA extremely well placed within the expanding class of multi‐agonist incretin peptide therapeutics, that includes dual/triple incretin agonists such as tirzepatide and retatrutide [56]. Importantly, apelin differs fundamentally from GIP, glucagon and other related islet‐intestinal derived peptide hormones that tend to be incorporated into GLP‐1 based hybrid peptides, being an adipokine and possessing strong insulin‐sensitising and islet‐preserving actions [24, 57] as well as key cardiovascular benefits [17]. Together this innovative strategy, that combines incretin and adipokine receptor‐mediated actions within a single molecule, offers a broader therapeutic footprint than current clinically available incretin agonists, as well as related developmental drugs [58]. Collectively, these studies provide clear proof‐of‐principle for GLP‐1/apelin hybrid peptides as a promising direction for next‐generation obesity and diabetes therapeutics, and future work employing sustained administration schedules will help further delineate clinical potential.

## Author Contributions

A.S., N.I. and F.P.M.O'H contributed to the overall concept, experimental design and interpretation of the data. A.S., S.L.C., E.K.‐K. and E.S.P. performed the experimental work and contributed to validation, formal analysis and visualisation of the data. All authors contributed to the writing of the manuscript and approved the final version.

## Funding

This work was supported by Invest Northern Ireland, Proof‐of‐Concept grant (PoC 825). The Northern Ireland Department for Education, PhD studentship.

## Disclosure

N.I. and F.P.M.O'H. are named on patents filed by Ulster University for exploitation of incretin‐based drugs and other peptide therapeutics, and are also shareholders in the Ulster University spin‐out company Dia Beta Labs Ltd.

## Conflicts of Interest

The authors declare no conflicts of interest.

## Data Availability

All data used to support the findings of this study are available from the lead scientist (F.P.M.O'H) upon request.
